# Infants Are Exposed to Human Milk Oligosaccharides Already *in utero*

**DOI:** 10.3389/fped.2018.00270

**Published:** 2018-10-02

**Authors:** Audra Wise, Bianca Robertson, Biswa Choudhury, Samuli Rautava, Erika Isolauri, Seppo Salminen, Lars Bode

**Affiliations:** ^1^Department of Pediatrics, University of California, San Diego, La Jolla, CA, United States; ^2^Rady Childrens Hospital San Diego, San Diego, CA, United States; ^3^Glycotechnology Core, Glycobiology Research and Training Center, University of California, San Diego, La Jolla, CA, United States; ^4^Department of Pediatrics, University of Turku and Turku University Hospital, Turku, Finland; ^5^Functional Foods Forum, Faculty of Medicine, University of Turku, Turku, Finland; ^6^Larsson-Rosenquist Foundation Mother-Milk-Infant Center of Research Excellence, University of California, San Diego, La Jolla, CA, United States

**Keywords:** human milk oligosaccharide, pregnancy, amniotic fluid, microbiome, immune system, breast milk

## Abstract

Human milk oligosaccharides (HMOs) are complex carbohydrates that are highly abundant in and, in their complexity, unique to human milk. Accumulating evidence indicates that exposure to HMOs in the postnatal period affects immediate as well as long-term infant health and development. However, studies reported in the 1970s showed that HMOs already appear in maternal urine and blood during pregnancy and as early as the first trimester. In this pilot study we aimed to determine whether or not HMOs also appear in amniotic fluid. We enrolled women during pregnancy and collected their urine and amniotic fluid at birth as well as their milk 4 days postpartum. We analyzed the samples by high-performance liquid chromatography (HPLC) and mass spectrometry and identified several HMOs including 2′-fucosyllactose, 3-fucosyllactose, difucosyllactose, and 6′-sialyllactose to be present in different relative abundancies in all three tissues. This is the first report that HMOs appear in amniotic fluid and that the fetus is already exposed to HMOs *in utero*, warranting future research to investigate the immediate and long-term implications on fetal and infant health and development.

## Introduction

Human milk oligosaccharides (HMOs) are a group of complex carbohydrates that are highly abundant in human milk but not in infant formula (1). Most recently, two of the more than 150 HMOs naturally occurring in human milk have been approved for food use in the European Union and the US and are being added to some infant formula (2–4). Therefore, current research focuses on the short- and long-term consequences for health and development when infants are exposed to the complex mixture of HMOs through breastfeeding and individual HMOs through formula-feeding.

Accumulating evidence demonstrates that HMOs are human milk prebiotics that serve as metabolic substrates for specific and potentially beneficial microbes (5). HMOs are also antimicrobials with direct bacteriostatic or bactericidal effects or antiadhesives that mimic glycocalyx structures on epithelial cell surfaces and serve as soluble decoy receptors to block the attachment of potentially pathogenic microbes (1, 6, 7). The combined prebiotic, antimicrobial, and antiadhesive effects of HMOs contribute to shaping the infant gut microbiome early in life, reducing the risk of acute diseases including infectious diarrhea or necrotizing enterocolitis (8–10), and potentially also that of non-communicable diseases such as asthma and allergic diseases (11, 12), diabetes and obesity (13, 14). In addition, HMOs also modify immune system maturation and responses—both dependent and independent of the microbiome (15, 16). Thus, exposure to HMOs in the post-partum period has both immediate as well as long-term implications for infant health and development.

Exposure to HMOs however, may already begin long before birth. Already in the 1970s, Hallgren et al. applied exchange chromatography and gas-liquid chromatography and reported that HMOs appear not only in human milk, but also in the maternal circulation and urine as early as at the end of the first trimester (17). Taking these findings and recent demonstration suggesting that the mother may provide the inoculum of microbial colonization already *in utero* (18, 19), we aimed to determine whether or not HMOs also appear in amniotic fluid, which had not been studied before, and reach the growing fetus before birth.

## Materials and methods

The study was conducted at the University of California San Diego and approved by the University's Institutional Review Board. We enrolled 48 women during pregnancy with the goal to collect their urine before delivery, their amniotic fluid during planned c-section or after spontaneous rupture of membrane, as well as their milk 4 days after birth. All samples were collected in sterile cups and stored at −20°C until analysis. Out of the enrolled 48 women, eight not only provided urine and milk, but also amniotic fluid samples that appeared clear and were free of blood (pink samples) or meconium (turbid samples). The mean gestational age for these eight subjects was 39 weeks (minimum: 38 weeks; maximum: 39 weeks and 5 days). Two of the eight deliveries were c-sections, two were vaginal delivery. Oligosaccharides from these eight sample sets were isolated by solid phase extraction chromatography over C18 and carbograph, labeled with 2-aminobenzamide (2AB), and analyzed by HPLC with fluorescence detection. In addition and to obtain a confirmation of HMOs in the sample, oligosaccharides were partially methylated, premixed with sDHB matrix, spotted on Matrix-Assisted Laser Desorption/Ionization (MALDI) plates, and analyzed by MALDI Time-of-Flight (TOF) (Autoflex, Bruker). The mass spectral data was acquired in positive, reflectron mode. To obtain structural details on specific oligosaccharides, samples were dissolved in methanol containing 1% formic acid and directly injected into a Linear Trap Quadropol (LTQ)-Orbitrap Discovery mass spectrometer (Thermo Scientific). The spectral data was collected on positive mode and selected ions were further fragmented to obtain ms/ms data.

## Results

**Figure 1:**
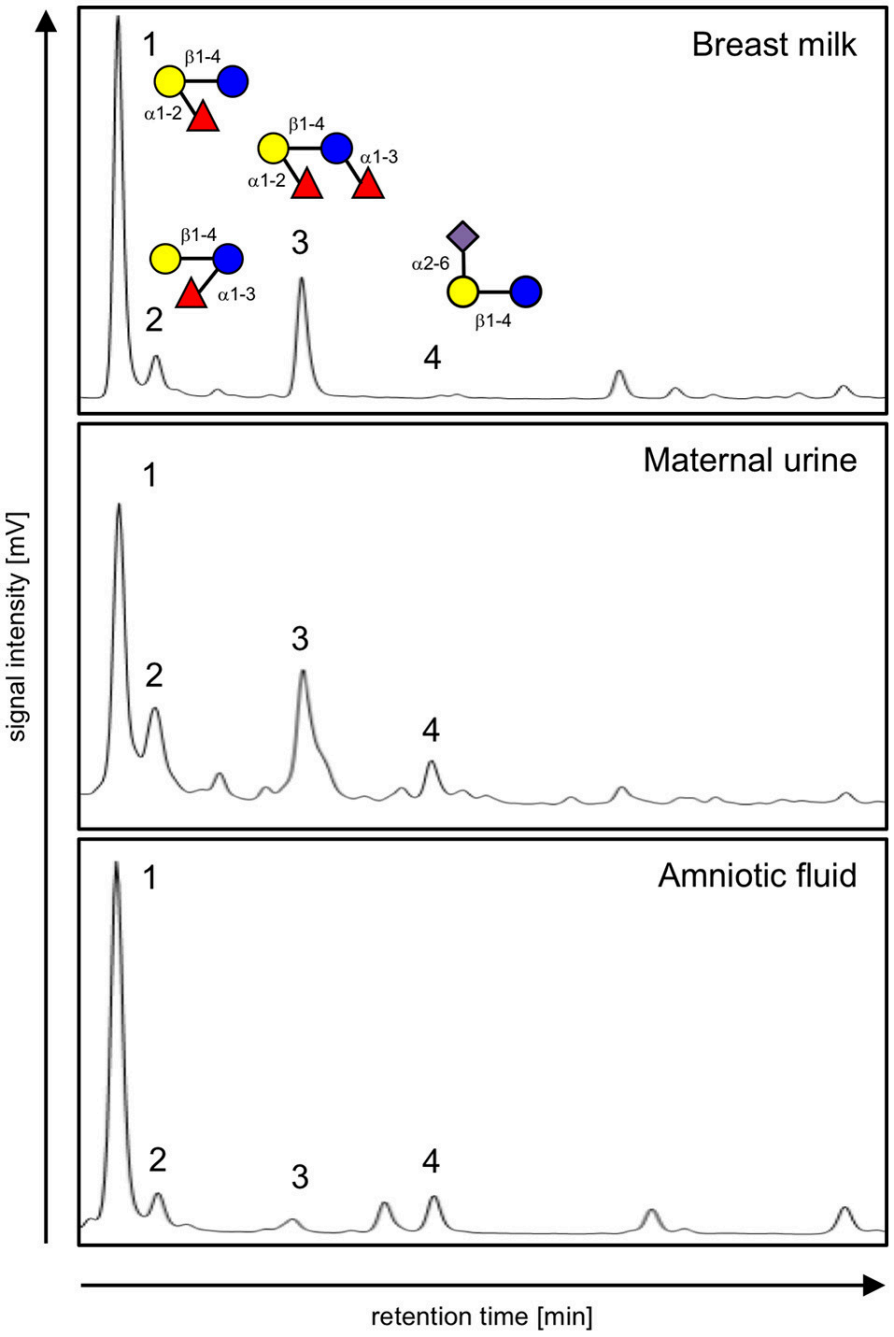
HPLC chromatograms of 2AB-labeled oligosaccharides isolated from breast milk (top), urine (middle), and amniotic fluid (bottom) from the same subject. Peaks are identified as (1) 2′-fucosyllactose, (2) 3-fucosyllactose, (3) difucosyllactose, and (4) 6′-sialyllactose based on standard retention times as well as offline mass spectrometric analysis. Oligosaccharide composition is shown in the top panel with glucose (blue circle), galactose (yellow circle), fucose (red triangle) and sialic acid (purple diamond) as monosaccharide building blocks.

**Figure 2:**
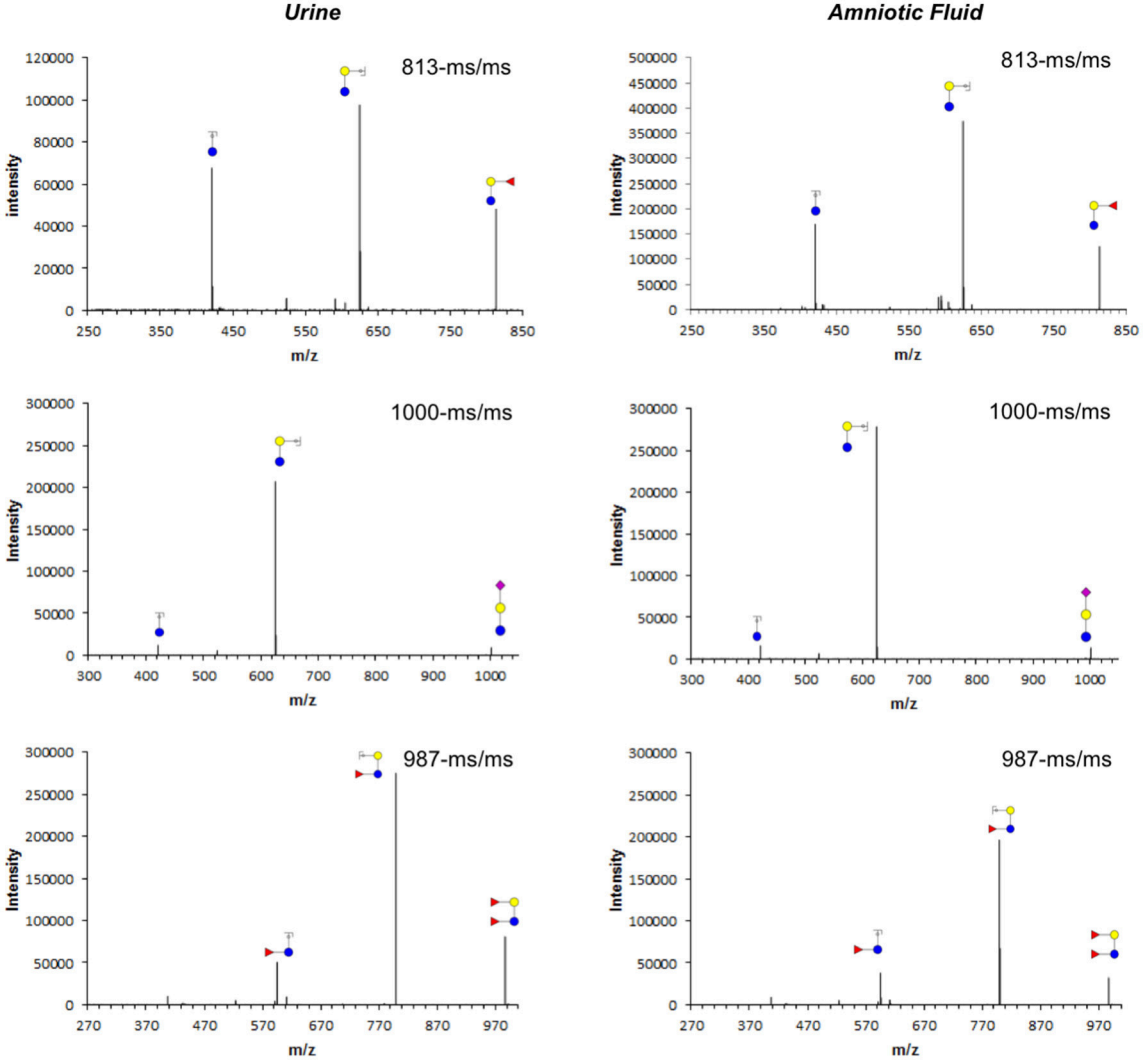
Tandem mass fragmentation of selected ions from 2-AB labeled per-O-methylated oligosaccharides isolated from maternal urine (left) and amniotic fluid (right). Cartoon structures of Y-type fragments are shown for parent mass 813 (2′-fucosyllactose, top), 1,000 (3′-sialyllactose, middle), and 987 (difucosyllactose, bottom) with glucose (blue circle), galactose (yellow circle), fucose (red triangle), and sialic acid (purple diamond) as monosaccharide building blocks.

The combination of HPLC (Figure 1) and offline mass spectrometry (Figure 2) identified four HMOs not only in milk, but also in maternal urine and in amniotic fluid. However, their relative abundance differs between sample types, suggesting differential transport across epithelial barriers in the mammary gland, kidneys, or placenta or differential degradation in or on the way to the different tissues. For example, 2′-fucosyllactose (2′FL) is the dominant oligosaccharide in all three sample types. 3-fucosyllactose (3FL) is more abundant in maternal urine than in milk and amniotic fluid. Difucosyllactose (DFLac) is more abundant in milk and urine than in amniotic fluid. 6′-sialyllactose (6′SL) is more abundant in urine and amniotic fluid and less in milk. Several other oligosaccharides with higher retention times and molecular weights can be detected in all three sample types, but structure elucidation in urine and amniotic fluid samples was not conclusive due to sensitivity issues.

## Discussion

This is the first report that HMOs appear in amniotic fluid and that the fetus is already exposed to HMOs *in utero*. Here, HMOs may have similar effects to what has been described for the post-partum period. If amniotic fluid is indeed not sterile as suggested (5), HMOs may act as prebiotics and contribute to shaping the amniotic fluid microbiome and set the stage for the developing infant microbiome post-partum, which may affect life-long health and disease risk. HMOs in maternal urine and amniotic fluid may also serve as antimicrobials and antiadhesives and help fight infections and inflammation, reducing the risk of chorioamnionitis and preterm delivery.

Unlike glycans that are part of glycoproteins or glycolipids (20), all HMOs carry lactose at the reducing end (1). Thus, mass spectrometric analysis can easily distinguish HMOs from any other non-HMO glycans or glycan fragments, proving unequivocally HMOs indeed appear in amniotic fluid. While we can clearly detect HMOs in amniotic fluid, we are not comfortable reporting absolute concentrations solely based on our HPLC and MS analysis.

While HMOs appear in maternal urine as early as at the end of the first trimester (17), our pilot study only analyzed amniotic fluid collected at birth and was not designed to assess how early HMOs appear in amniotic fluid. Additional research will be required to investigate when HMOs start to appear in amniotic fluid, whether or not the composition changes over time, and how much the composition varies between women.

Nonetheless, the observation that HMOs appear in amniotic fluid is novel and warrants future clinical research to identify associations and cause-and-effect relationships between amniotic fluid HMO composition and clinical outcome measures, which may lay the foundation for future applications with the aim to alter and improve amniotic fluid HMO amount and composition to impact maternal and infant health at the perinatal-neonatal continuum with potential life-long consequences.

## Ethics statement

This study was carried out in accordance with the recommendations of the University of California, San Diego Institutional Review Board guidelines with written informed consent from all subjects. All subjects gave written informed consent in accordance with the Declaration of Helsinki. The protocol was approved by the Institutional Review Board.

## Author contributions

SR, EI, SS, and LB conceived the idea. AW recruited women and collected samples. AW, BR, and BC analyzed the samples. SR, EI, SS, and LB wrote the manuscript.

### Conflict of interest statement

The authors declare that the research was conducted in the absence of any commercial or financial relationships that could be construed as a potential conflict of interest.
